# Multimodal deep learning applied to classify healthy and disease states of human microbiome

**DOI:** 10.1038/s41598-022-04773-3

**Published:** 2022-01-17

**Authors:** Seung Jae Lee, Mina Rho

**Affiliations:** 1 Department of Computer Science, Hanyang University, Seoul, Korea; 2 Department of Biomedical Informatics, Hanyang University, Seoul, Korea

**Keywords:** Genome informatics, Machine learning

## Abstract

Metagenomic sequencing methods provide considerable genomic information regarding human microbiomes, enabling us to discover and understand microbial diseases. Compositional differences have been reported between patients and healthy people, which could be used in the diagnosis of patients. Despite significant progress in this regard, the accuracy of these tools needs to be improved for applications in diagnostics and therapeutics. MDL4Microbiome, the method developed herein, demonstrated high accuracy in predicting disease status by using various features from metagenome sequences and a multimodal deep learning model. We propose combining three different features, i.e., conventional taxonomic profiles, genome-level relative abundance, and metabolic functional characteristics, to enhance classification accuracy. This deep learning model enabled the construction of a classifier that combines these various modalities encoded in the human microbiome. We achieved accuracies of 0.98, 0.76, 0.84, and 0.97 for predicting patients with inflammatory bowel disease, type 2 diabetes, liver cirrhosis, and colorectal cancer, respectively; these are comparable or higher than classical machine learning methods. A deeper analysis was also performed on the resulting sets of selected features to understand the contribution of their different characteristics. MDL4Microbiome is a classifier with higher or comparable accuracy compared with other machine learning methods, which offers perspectives on feature generation with metagenome sequences in deep learning models and their advantages in the classification of host disease status.

## Introduction

Since the introduction and application of next-generation sequencing technologies to human genomes and their microbiomes, many researchers have exploited its application in disease diagnostics and therapeutics. Genetic variation in the human genome is an important feature for diagnosing diseases, such as cancer^1,2^. In the past few decades, advanced metagenomic sequencing methods have allowed research on the human microbiome to find pathological relationships between bacterial composition and functions with the disease. The Human Microbiome Project (HMP) has sequenced more than 700 samples of microbial communities from different body sites of healthy individuals^3^. Subsequently, the Integrated Human Microbiome Project (iHMP) has collected microbiome samples from three different microbiome-associated dysbiosis conditions^4^.

With the increasing amount of metagenome sequencing data, more studies have characterized profiles of the human microbiome for a deeper analysis. Traditionally, alignment-based methods, such as MetaPhlAn^5^, have been widely used for taxonomy profiling. Despite their algorithmic differences, all alignment-based programs rely on the current databases of bacterial genomes. To address such problems, time-efficient, alignment-free methods, such as Kraken^6^ and CLARK^7^, have been applied to profiling^8^, which assign taxonomic labels to metagenomic sequences based on the k-mer frequency. In addition, microbial functions have also been analyzed to understand the physiology of the disease. Using well-curated databases, such as KEGG^9,10^, COG^11,12^, and subsystems^13^, metabolic functions are annotated for specific microbiomes using various algorithms^14^.

In the skin, mouth, nose, and digestive tract of humans, the microbiota is composed of diverse species of microorganisms in different proportions, which can be meaningful indicators of the disease status^15^. Recent studies have used computational methods to profile microbial compositions in samples to differentiate between healthy and disease states^16–18^. For example, the gut microbiome composition of patients with inflammatory bowel disease (IBD) is different from that of healthy people^19–22^. Liver disorders have been studied to reveal a correlation with altered gut microbiome^17,23^. Studies of the human gut microbiota have shown that the interplay between microbes and the host is associated with various medical factors^24^.

For the classification of host health states regarding the microbiome, machine learning methods were applied using amplicon sequencing data^25^. Conventionally, operational taxonomic unit (OTU) representations are commonly used as input features for neural networks. MetaDP uses 16S sequencing data to generate OTU tables that contain information about microbial composition and diversity as features for the SVM-based prediction^26^. MicroPheno uses k-mer distribution features in body site identification and Crohn’s disease prediction, which is more accurate and time-efficient than using conventional features^27^. MetaNN overcomes the overfitting problem by applying data augmentation and dropout training techniques to multilayer perceptron and convolutional neural network models^28^. Most of the classifiers were developed based on amplicon sequencing data, which is cost-efficient but provides limited information that is captured from the comprehensive microbiome. As being already reported, microorganisms in the human body have numerous significant intrinsic features for diagnosing diseases^29^, and even the same species may differ genetically and perform different functions^30^.

In this study, we developed a deep learning model called MDL4Microbiome to classify disease status using the features extracted from microbiome sequencing data. Our classifier was built using a multimodal neural network based on the compositional and functional aspects of the human microbiome, which achieved the higher or comparable accuracies of 0.98, 0.76, 0.84, and 0.97 in predicting patients with IBD, Type 2 diabetes (T2D), liver cirrhosis (LC), and colorectal cancer (CRC), respectively.

## Methods

### Data preparation and preprocessing

All methods were performed in accordance with the relevant guidelines and regulations. Four datasets were used to train and validate the classifier. The first set was patients with IBD and healthy individuals, the second was patients with T2D and healthy individuals, the third was patients with LC and healthy individuals, and the fourth set was patients with CRC and healthy individuals. The IBD dataset was downloaded from the NIH Common Fund’s HMP Program (100 controls and 100 IBD patients)^3,4^. The T2D dataset was downloaded from NCBI Sequence Read Archive under accession numbers SRA045646 and SRA050230 (47 controls and 101 T2D patients)^31^. The LC dataset was downloaded from the European Nucleotide Archive (ENA) under the accession number ERP005860 (83 controls and 94 LC patients)^17^. The CRC dataset was downloaded from the ENA under the accession number PRJEB27928 (60 controls and 59 CRC patients)^32^ . Each dataset consisted of 200, 148, 177, and 119 samples of gut microbiome sequencing data from healthy individuals and patients with IBD, T2D, LC, and CRC, respectively. Additional information on the samples is provided in Supplementary Table S1.

For each downloaded raw sample, paired-end sequencing reads were trimmed for quality control. Low-quality reads (Phred quality score < 20) were removed using Sickle^33^. All reads containing Ns in their sequences were also removed. Taking into account the technological imperfections in extracting gut microbiome, host contaminations were removed by mapping reads to the UCSC human reference genome (GRCh37, hg19, established in February 2009) using Bowtie (ver. 2.3.4.1)^34^. Throughout the mapping results, reads with a mismatch and soft-clip length under 10% and 30% of the read length were considered as human contaminations and removed.

### Generation of feature sets

**Figure 1:**
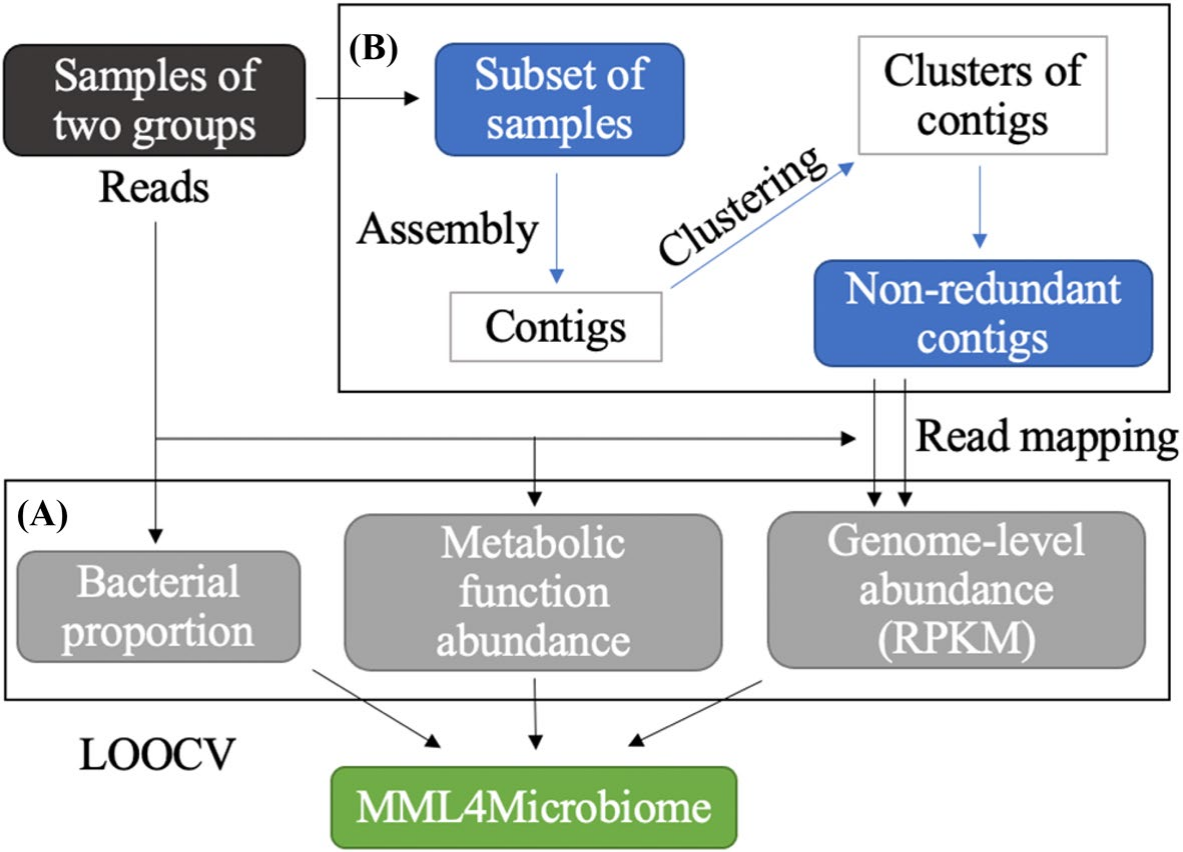
Schematic diagram of MDL4Microbiome. **(A)** Three methods were used to generate different types of features, viz., conventional taxonomic profiles, metabolic functional features, and genome-level abundance. Different features are fed into the multimodal deep learning model. The model was evaluated by the leave-one-out cross-validation method. **(B)** Specific steps of extracting non-redundant contigs of known and unknown microorganisms. A subset of samples is selected randomly. After contigs are assembled with the reads of the selected samples, they are clustered to collect a set of non-redundant representative contigs. Entire sample reads are mapped to non-redundant contigs to measure the relative abundance of genomic fragments.

The proposed classifier was constructed and trained using the essential information of the microbiome data. Three different approaches were used for extracting features in this study, i.e., two for the relative abundance of microbial composition and one for functional characteristics. MDL4Microbiome combined all three features in a multimodal model (Fig. 1).

The conventional composition profile was generated at seven different taxon ranks from phylum to species using MetaPhlan (version 2.1.0)^5^. MetaPhlan was performed with the default options except the ignore flags (–ignore-archaea, –ignore-eukaryotes, –ignore-viruses), leaving only bacteria in the profiles. Any genus and species that appeared in only one sample were removed because they could be sample-specific. For the IBD, T2D, and LC datasets, a total of 327, 406, and 316 species were identified, respectively. The number of taxa at each rank, which is the number of features in the modal for the conventional taxonomic profile, is provided in Table 1. Each feature value is the proportion of each taxon. Proportion values were log-transformed before feeding them into neural network models^35^.

**Table 1:** Number of input features in the IBD, T2D, and LC datasets.

Features		IBD	T2D	LC	CRC
Taxonomic composition	Phylum	12	11	11	11
	Class	20	19	18	17
	Order	26	32	27	26
	Family	52	62	54	52
	Genus	116	141	121	122
	Species	327	388	361	313
Genomic contigs^a^	2 Refs	27	50	23.7	69
	40 Refs	279.3	366	207.3	479.3
Functional proportion		6,147	5,333	7,381	7,220

^a^For genomic features, an average of three runs conducted with different representative samples was calculated.

Features of genome-level relative abundance were generated to consider genomic variation information that taxonomic profiles may not include. We used the term “genome-level” to indicate the genomic regions that are conserved in a group of strains. The abundance of such specific genomic regions was extracted as a type of feature. Therefore, reference samples were randomly selected from all the training samples in the dataset. Performance was evaluated according to the number of reference samples used (see “Results” section). To construct contigs, paired-end reads of the reference samples were assembled using MEGAHIT (ver. 1.1.3)^36^. Of all contigs from the reference samples, contigs longer than 5000 bp were retained. Binning was performed on the contigs to retain non-homologous contigs, which improved the computational time with comparable accuracy performance (see “Results” section). The contigs were binned with MetaBAT (ver. 2.15)^37^. All parameters were set as default with no depth file as an option when running MetaBAT. The longest contig in each bin was selected and gathered as non-redundant representative contigs for feature generation.

The reads in each sample were mapped to non-redundant contigs using Bowtie (ver. 2.3.4.1)^34^. For each pair of a sample and non-redundant contig, the values of reads per kilobase per million mapped reads (RPKM) were calculated as follows:

$$RPKM({sample}_{i},\,{contig}_{j})=\frac{number\, of\,mapped\, reads\, in\, {sample}_{i} *{10}^{3}*{10}^{6}}{number\, of \, reads\, in\, {sample}_{i}* length\, of\, {contig}_{j}}$$

The RPKM values represent the relative abundance of genomic fragments. Contigs with coverage under 70% were disregarded since such genomic fragments might not exist in a certain sample. The coverage of contig j in sample i was calculated as follows:

$$Coverage\,\left({sample}_{i}, {contig}_{j}\right)= \frac{length\, of\, {contig}_{j}\, covered\, by\, reads\, in\, {sample}_{i}}{length\, of\, {contig}_{j}}$$

Log transformation was applied to genomic features before feeding them into neural networks.

An abundance of metabolic functions was generated as a feature. Using 14,785 ortholog gene clusters provided by KEGG (ver. 54)^9,10^, metagenomic sequencing reads were searched using DIAMOND (ver. 0.9.14)^38^ with the following parameters: percent identity and query coverage cutoffs were set as 50% and 50%, respectively; e-value cutoff was 1.0e−10. For reads, RPKM values were calculated for all KEGG ortholog (KO) proteins that matched at least one read in the dataset. These values were summed into functional categories and normalized by gene length.

### Construction of multimodal deep learning model

**Figure 2:**
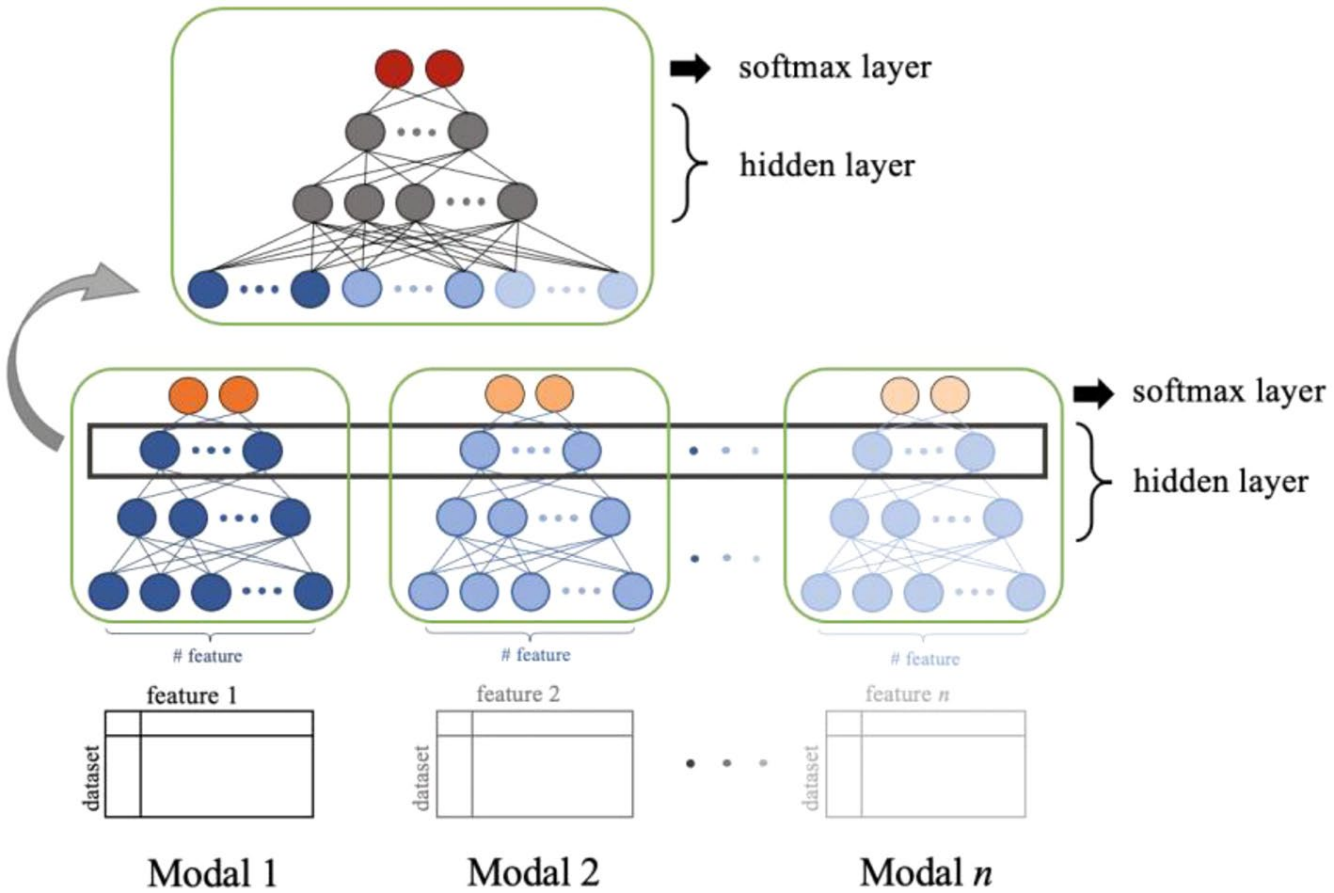
Architecture of multimodal deep learning model. A multimodal deep learning model aims for combining features from different modalities. Each feature generated by different methods is first fed to the classifier. The nodes of the last hidden layer are considered as embedded representations of each feature. Embedded representations are concatenated into a new shared representation inheriting original features. Combined feature representation is fed to the classifier for final classification.

A multimodal deep learning model was used to combine different types of features in MDL4Microbiome (Fig. 1). More specifically, species-level profiles, genomic features generated with 40 reference samples, and metabolic functional features generated using the KEGG database were used. The architecture of the model is shown in Fig. 2. Different features were fed into a separate supervised deep neural network model. The last hidden layer represents the embedded representations of each feature. Combining each representation, we obtained a new shared representation that inherits original features from different modalities. For comparison, individual features were trained using simple deep neural network classifiers.

Multimodal deep learning models and simple deep neural network models were implemented in Python (version 3.6.9) for the evaluation. Keras (version 2.3.1), Python deep learning API, was used to build and compile all neural network models. Each layer was created as a dense layer with a fully connected configuration. Models were created and compiled for multiclass classification. The activation function for the output layer was set to the softmax function. The activation functions for all the other layers were rectified linear unit (ReLU) functions. For compilation, the Adam optimizer was used with default settings of learning rate = 0.001, beta_1 = 0.9, and beta_2 = 0.999.

The number of nodes in each hidden layer and the number of hidden layers were considered in the design of the architecture of the models. The performances of the models with various structures of hidden layers are presented in Supplementary Table S2. There was no significant difference with an increase in the number of nodes and hidden layers. The execution time increased as the number of nodes and hidden layers increased. For time efficiency and fair comparison between features, three hidden layers consisting of 200, 100, and 50 nodes were used for taxonomic and genomic features. For functional features, regarding the bigger feature sets, the number of hidden layers were the same, but were implemented with 500, 100, and 50 nodes. For the final classifier in multimodal learning models, only two hidden layers consisting of 50 and 25 nodes were used.

### Performance evaluation

Model evaluation was performed using the leave-one-out cross-validation (LOOCV) method. LOOCV is the case of k-fold cross-validation, where k is the number of samples. In our evaluation process, one sample was excluded in both stages of training, i.e., feature embedding and final classification, and used in testing. Accuracy, precision, and recall were used to evaluate the performance as follows:

$${\text{Accuracy}}= \frac{TP+TN}{TP+FP+TN+FN}$$

$${\text{Precision}}= \frac{TP}{TP+FP}$$

$${\text{Recall}}= \frac{TP}{TP+FN}$$

where TP, FP, TN, and FN represent true positive, false positive, true negative, and false negative, respectively. LOOCV was conducted five times and averaged for the evaluation. For genomic features, three runs were conducted by randomly selecting new reference samples in each iteration. For the IBD and T2D datasets, macro- and micro-averaged accuracies were the same because both datasets included the same number of positive and negative samples. For the LC dataset, the number of positive and negative samples were similar, resulting in no significant difference between macro- and micro-average performances.

To evaluate the performance, five traditional machine learning models (i.e., random forest (RF)^39^, extreme gradient boosting (XGBoost)^40^, principal component regression (PCR), lasso regression^41^, and support vector machine (SVM)^42^ were used. All the traditional models were implemented in Python (version 3.6.9). For RF, PCR, lasso regression, and SVM, scikit-learn library (version 0.24.2) of python machine learning package, was used. For XGBoost, the xgboost 1.5.0-dev library was used.

## Results

### Performance evaluation with various model architectures and parameters

To evaluate the accuracy with respect to model architectures, four different models were constructed using different features. We measured the accuracy using three large-scale metagenome sequencing data: the IBD, T2D, and LC datasets. With the IBD dataset, precision, recall, and accuracy were 0.97, 0.98, and 0.98, respectively. With the T2D dataset, values of 0.80, 0.86, and 0.76, respectively, were achieved, and with the LC dataset, values of 0.86, 0.83, and 0.84, respectively, were achieved. Lastly, values of 0.99, 0.94, and 0.97 were achieved with the CRC dataset. Notably, multimodal neural networks achieved the best accuracy for all four datasets, compared to simple DNN classifiers with individual feature types (Table 2 and Supplementary Table S3).

**Table 2:** The performance of four different model architectures.

Features		IBD			T2D			LC			CRC		
		P	R	A	P	R	A	P	R	A	P	R	A
Taxonomic composition	Phylum	0.69	0.71	0.7	0.66	0.80	0.58	0.77	0.57	0.68	0.57	0.41	0.55
	Genus	0.87	0.87	0.87	0.76	0.77	0.68	0.83	0.82	0.81	0.66	0.66	0.66
	Species	0.91	0.87	0.89	0.75	0.82	0.69	0.86	0.78	0.81	0.71	0.67	0.70
Genomic contigs^a^	2 Refs	0.89	0.85	0.87	0.72	0.80	0.65	0.71	0.73	0.70	0.62	0.54	0.61
	40 Refs	0.94	0.92	0.93	0.77	0.85	0.72	0.81	0.8	0.79	0.84	0.75	0.81
Functional proportion		0.77	0.85	0.80	0.74	0.86	0.70	0.74	0.88	0.77	0.98	0.90	0.94
All combined (multimodal)		0.97	0.98	0.98	0.80	0.86	0.76	0.86	0.83	0.84	0.99	0.94	0.97

^a^For each experiment, LOOCV was used to calculate the precision, recall, and accuracy. Five simulation runs were performed, and the values were averaged over five runs.

*P* precision, *R* recall, *A* accuracy.

When using a conventional genus-level profile as input features, the accuracies of the classifier were 87.3%, 67.6%, 81.4%, and 66.4% for the IBD, T2D, LC, and CRC datasets, respectively, with the same probability threshold of 0.5. For the species-level profile, the accuracies were 89.4%, 68.9%, 81.5%, and 70.0% for IBD, T2D, LC, and CRC datasets, respectively. Using the probability threshold of 0.5, the model with genome-level variation achieved accuracies of 92.9%, 72.3%, 79.0%, and 80.7% for the IBD, T2D, LC, and CRC datasets, respectively, with 40 reference samples selected in the feature generation process. Using metabolic functional features, the accuracies were 79.5%, 70.3%, 77.4%, and 94.1% for IBD, T2D, LC, and CRC datasets, respectively.

**Figure 3:**
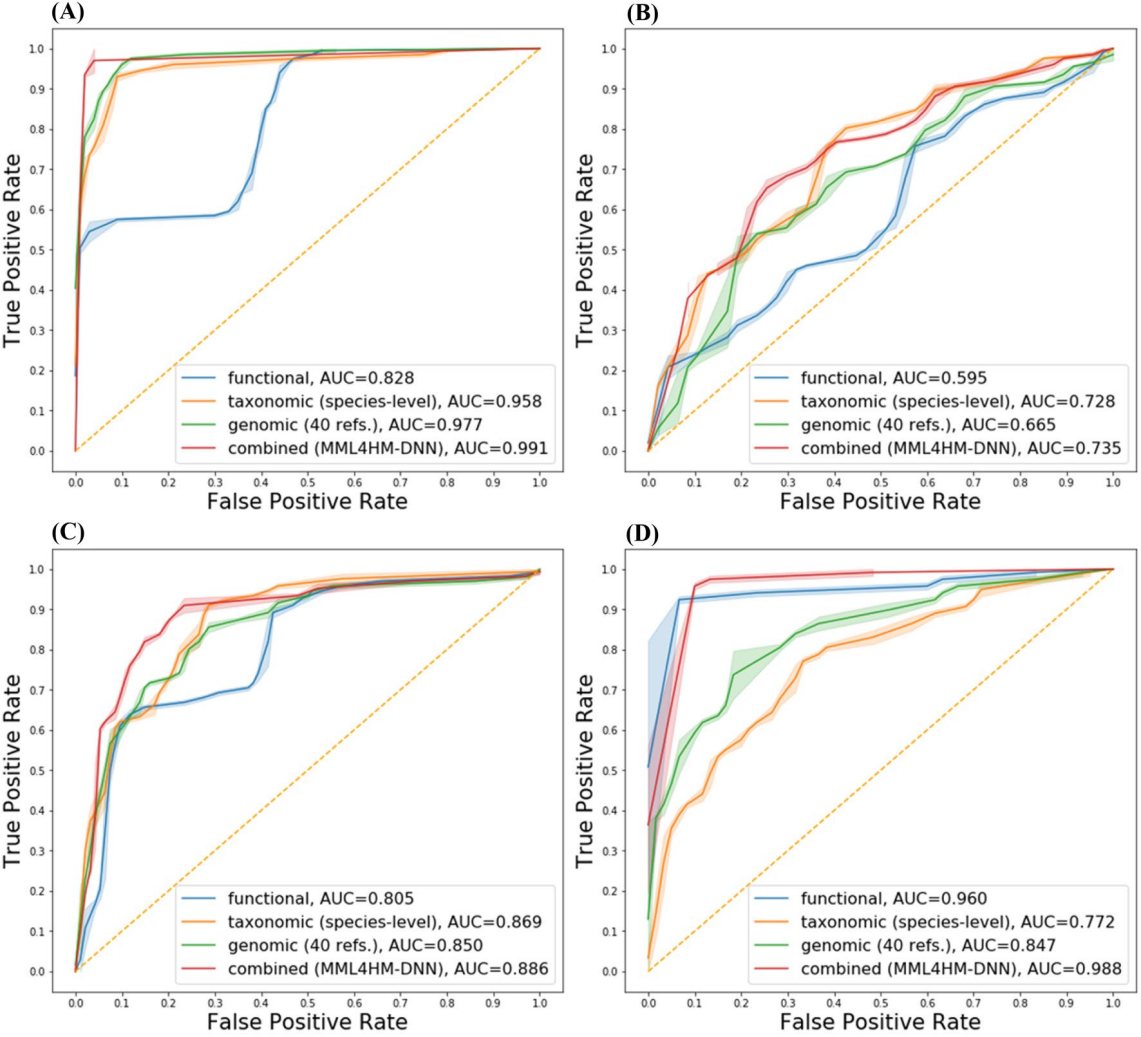
ROC curves and AUCs for MDL4Microbiome with each feature and all features combined. For ROC curves, thresholds were selected as the means between any two consecutive values observed in the data. ROC curves and AUCs for the (A) IBD, (B) T2D, (C) LC, and (D) CRC datasets.

**Figure 4:**
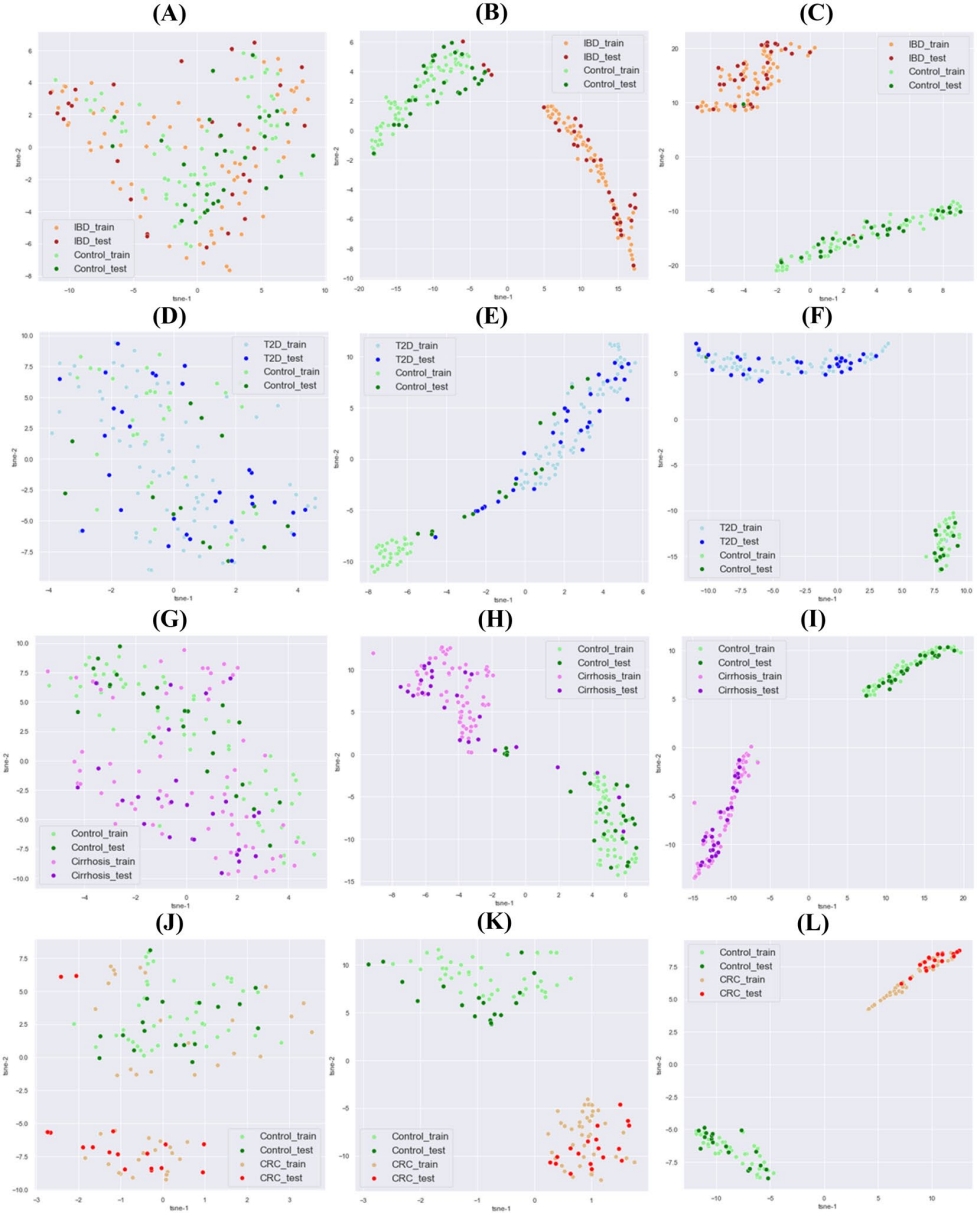
Visualizations of the IBD, T2D, and LC datasets before and after training using t-SNE. Each column, (**A–C**), (**D–F**), (**G–I**), and (**J–L**) was generated with IBD, T2D, LC, and CRC datasets, respectively. (**A,D,G,J**) are from the data before training MDL4Microbiome with all features combined (simply concatenated). (**B,E,H,K**) are from the data in the last hidden layer when the classifier was trained with 70% of the dataset (as light colors). The remaining 30% retained for testing were predicted using the classifier (as dark colors). (**C,F,I,L**) are the result of one-fold of LOOCV. All samples, except for one, in the dataset were used for training, and all samples were included in prediction for visualization.

As shown in the receiver operating characteristic (ROC) curves and area under curve (AUC), the multimodal neural network showed better performance compared to the neural network with single type of feature (Fig. 3). In particular, for IBD, LC, and CRC datasets, the ROC curves and AUC values improved dramatically when combining the features and using a multimodal deep learning model. To analyze how well the decision boundary was set with combined features compared to each of the features, the training and testing datasets were plotted using t-SNE. Three different views were observed, viz., data distribution before training; with split training and testing data with a ratio of 7:3; after training in one of the folds in LOOCV (Fig. 4). Before training, distribution of samples with the raw feature values, obtained through simple concatenation of three different features, did not show a clear separation (Fig. 4A,D,G,J). When the data were divided into two groups for training and testing at a ratio of 7:3, the data from two different phenotypic groups were relatively well-separated to achieve higher accuracy. When the testing data were evaluated by combined features, the testing data were well-aligned with training data from the two different groups (Fig. 4B,E,H,K). After training with all samples except for one in the dataset as part of the LOOCV, the datasets showed better separation (Fig. 4C,F,I,L), which may suggest that a larger amount of training data increases the accuracy of this LOOCV evaluation process.

### Effects of different features on the performance

**Figure 5:**
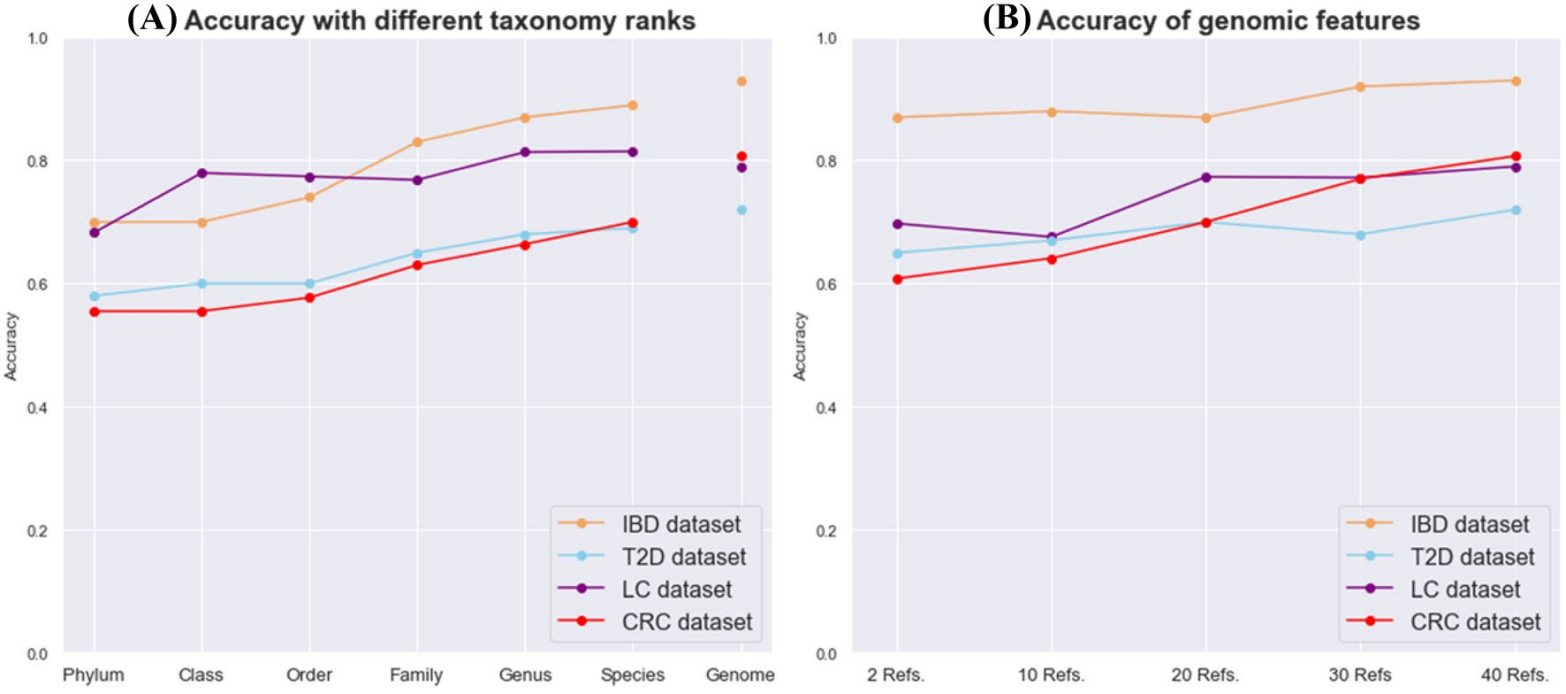
Accuracy with different levels of relative abundance features. **(A)** Accuracy of conventional taxonomic features from phylum-level to species-level and genomic features generated with 40 reference features for comparison. **(B) **Accuracy of genome-level features with different numbers of reference samples. Each data point is an average accuracy of three runs conducted with different reference samples. Each run represents an average accuracy of five iterations of LOOCV. Orange, blue, purple, and red lines correspond to the IBD, T2D, LC, and CRC datasets, respectively.

As the lower rank of taxonomy (i.e., from phylum to species) was used as profile features, the accuracy generally increased for all datasets (Fig. 5A). The phylum-level profile features had the lowest accuracy of 69.7%, 58.1%, 68.3%, and 55.5% whereas the species-level profile features had the highest accuracy of 89.4%, 68.9%, 81.5%, and 70.0% for the IBD, T2D, LC, and CRC datasets, respectively. The LC dataset showed a similar pattern to other datasets, except for a protruding point at the class-level. Moreover, for IBD, T2D, and CRC datasets, the genome-level variation features showed the highest accuracy of up to 92.9%, 72.2%, and 80.7%, which exceeded the accuracy of all taxonomic features. With the LC datasets, the genome-level variation features slightly decreased the accuracy.

When generating genomic features, the number of reference samples affects the accuracy (Fig. 5B). When there were more than a certain amount of reference samples (10 for the IBD and 20 for the CRC), the accuracy of genomic features surpassed the taxonomic proportions of all ranks (Fig. 5B). For the T2D dataset, with 20 and 40 reference samples, genomic features exceeded the taxonomic proportions of all ranks. This implies that a set of specific strains could be more associated with disease physiology than general taxonomy information on bacterial composition. Although both methods use relative abundance information generated based on metagenome sequencing data, genome-level variations achieved better performance than the conventional taxonomy profiles. We suspected that the lower accuracy of the LC dataset compared to that of the other two datasets resulted from the high diversity of samples within the group.

Although conventional composition profile features primarily consist of annotated taxa, genome-level variation uses relative abundance without taxonomic information. To confirm that genome-level relative abundance can provide more abundant taxonomic information, two samples of the gut microbiome from patients with IBD and healthy individuals were profiled by CAT^38,43,44^. As a result, 10% of the sequences could not determine their taxa at the genus level using CAT. Although this result does not represent the proportion of unknown taxa, these results imply that certain amounts of genomic sequences are not taxonomically annotated; thus, our approach of using the relative abundance of genomic sequences could provide more informative features for characterizing and comparing the microbiomes of different disease states.

When generating genomic features, the representative contigs were gathered with the longest sequences in each cluster (see Method section). We also checked whether the contigs selected from each cluster could represent each unique taxon. For this purpose, we analyzed the sequences in each cluster using CAT^38,43,44^, counting the number of contigs that were assigned to the same taxonomy. On average, 87.9% of the clusters had over 70% of contigs classified to the same taxon at the family and genus levels. Over 86% of the clusters had their longest sequences assigned to the dominant taxa in the cluster. This indicates that the clustering and selecting the longest contig from each cluster for representative contigs works properly to generate genomic features.

The classifier was also trained with metabolic function features obtained using the KEGG database (see “Methods” “Construction of multimodal deep learning model”). Using a probability threshold of 0.5, the model achieved an accuracy of 79.5%, 70.3%, 77.4%, and 94.1% for the IBD, T2D, LC, and CRC datasets, respectively (Table 2). Even though the accuracy with metabolic function features alone was lower than the compositional features (i.e., taxonomic, and genomic features) except for the CRC dataset, the combined features increased the performance in the multimodal model. This demonstrates that when classifying patients from healthy people, functions of microbiomes are still powerful features, and the potency may vary across diseases.

### Accuracy comparison with existing models

When the performance of the current model was compared to that of previous studies, our multimodal deep learning model with all combined features achieved higher or comparable performance. We could not find any classifier based on the whole metagenome sequencing data. MicroPheno^45^ and MetaNN^28^ utilized 16S rRNA gene sequencing data to predict host phenotypes. MicroPheno generated k-mer representation features and compared multiple classifiers, such as random forest (RF), SVM, and deep neural networks. For the Crohn’s disease dataset, the top micro- and macro-F1 scores were 0.76 and 0.75, respectively, using RF. MetaNN also compared several classifiers using an in-house data-augmentation method for taxonomy abundances. The highest micro- and macro-F1 scores were 0.84 and 0.78, respectively, for the IBD dataset using the MLP classifier with the dropout training technique.

Conventional machine learning models (i.e., RF, XGBoost, PCR, lasso regression, and SVM), were used for further comparisons (Table 3). For the input, three different features were concatenated into a single feature. For T2D and LC datasets, MDL4Microbiome outperformed all the other single models. In addition, an ensemble model was built with the combination of PCR, lasso regression, and SVM classifiers. The voting method was applied for the final prediction of the ensemble model.

**Table 3:** The accuracy of our model and conventional machine learning models.

	RF	XGBoost	PCR	lasso	SVM	Ensemble^a^	MDL4Microbiome
IBD	0.98	0.98	0.99	0.99	0.73	0.99	0.98
T2D	0.68	0.72	0.68	0.72	0.68	0.74	0.76
LC	0.81	0.82	0.83	0.82	0.82	0.84	0.84
CRC	0.94	0.94	0.95	0.96	0.87	0.94	0.97

^a^Ensemble of PCR, lasso, and SVM.

### Time complexity for feature generation

When reference samples are used in training, non-redundant sets of genomic sequences need to be selected as features because multiple samples have the same genomic sequences originating from the same taxon. The number of redundant sequences affects the amount of time required for mapping to generate features. From all contigs that were assembled from the reference samples, contigs of non-redundant sequences were obtained by clustering using MetaBAT^37^. The number of representative contigs reduced significantly after clustering (Supplementary Table S4). The amount of time consumed for mapping sequencing reads from the 200 IBD samples to 9,373 representative contigs (from one of the runs, two reference samples) was approximately 26.76 h (with 20 threads option in running Bowtie). However, when clustering was applied, the number of non-redundant sequences was reduced to 44, which took approximately 9.85 h (with the same options). The process for feature generation was approximately 2.72-times faster when the non-redundant set was used. As the number of reference samples increased, the reduction in time complexity increased.

## Conclusion

The gut microbiome is a collective set of microorganisms inside the human digestive tract and is a good indicator of human health. MDL4Microbiome showed higher or comparable accuracy for predicting the phenotypes of the hosts by combining features that were extracted on the basis of three different ways from metagenome sequencing data, i.e., on the basis of the conventional composition profiles, genome-level abundance, and metabolic functional abundance. Moreover, MDL4Microbiome achieved accuracies of 0.98, 0.76, 0.84, and 0.97 for the IBD, T2D, LC, and CRC datasets, respectively.

Compared to the taxonomy profiles for the microbiome, genome-level measurement of bacterial abundance could provide two advantages: first, the provision of a deeper level, supposedly, strain-level abundance information, and second, the provision of abundance information for unannotated taxa. Our method of generating genomic features achieved accuracies of 92.9%, 72.3%, and 80.7%, and the conventional profile-based classifier had accuracies of 89.4%, 69.9%, and 70.0% (in particular, species-level profile using MetaPhlAn) for the IBD, T2D, CRC datasets, respectively. When non-redundant contigs were extracted using binning before calculating the RPKM, the time required for feature generation decreased markedly. The process for feature generation (in particular, genomic features with two reference samples) was approximately 2.72-fold faster when binning was applied. Despite the advantages of our method, further studies are needed to identify unannotated species that contribute towards important features for diagnosing a disease. Metabolic function features were also evaluated. We showed that metabolic functions act as a significant feature for predicting disease states in the T2D dataset. In summary, the multimodal deep learning method allowed the combination of features of different aspects of microbiomes, resulting in an overall high accuracy of classifying host phenotypes.

## Supplementary Information

Supplementary Tables.

## Data Availability

The codes and the models in this article can be found at the public repository at https://github.com/DMnBI/MDL4Microbiome.

## Abbreviations

HMP: Human microbiome project
IBD: Inflammatory bowel disease
OTU: Operational taxonomic unit
LC: Liver cirrhosis
T2D: Type 2 diabetes
RPKM: Reads per kilobase per million mapped reads
KO: KEGG ortholog
ReLU: Rectified linear unit
LOOCV: Leave-one-out cross-validation
ROC: Receiver operating characteristic
AUC: Area under curve

## Code availability

Project name: MDL4Microbiome; Project home page: https://github.com/DMnBI/MDL4Microbiome; Operating system(s): Linux; Programming language: Python version 3.6.9; License: FreeBSD etc.
