# Supplementary material for: Multimodal deep learning applied to classify healthy and disease states of human microbiome
Source: Sci Rep. 2022 Jan 17;12:824. doi: 10.1038/s41598-022-04773-3 (PMC8763943; doi:10.1038/s41598-022-04773-3)
Supplement: Supplementary file 1 — Supplementary Tables. [file 41598_2022_4773_MOESM1_ESM.docx]

**Supplementary Table S1.** Table of all the sample IDs used in this paper. The first column presents dataset names, and the numbers between the parentheses represent the numbers of healthy and patient sample, respectively.

| **IBD (100, 100)** | **T2D (47, 101)** | **LC (83, 94)** | **CRC (60, 59)** |
| --- | --- | --- | --- |
| SRS011084 (Healthy) | SAMN00993239 (T2D) | SAMEA2581875 (HD-1) | CCMD10032470ST-11-0 (control) |
| SRS011134 (Healthy) | SAMN00993240 (T2D) | SAMEA2581876 (HD-10) | CCMD10191450ST-11-0 (control) |
| SRS011239 (Healthy) | SAMN00993241 (T2D) | SAMEA2581877 (HD-11) | CCMD11006829ST-21-0 (CRC) |
| SRS011271 (Healthy) | SAMN00993242 (T2D) | SAMEA2581878 (HD-12) | CCMD13071240ST-21-0 (CRC) |
| SRS011302 (Healthy) | SAMN00993243 (T2D) | SAMEA2581879 (HD-13) | CCMD13934959ST-21-0 (CRC) |
| SRS011405 (Healthy) | SAMN00993244 (T2D) | SAMEA2581881 (HD-14) | CCMD14479708ST-21-0 (CRC) |
| SRS011529 (Healthy) | SAMN00993245 (T2D) | SAMEA2581882 (HD-15) | CCMD15562448ST-11-0 (control) |
| SRS011586 (Healthy) | SAMN00791971 (Healthy) | SAMEA2581886 (HD-17) | CCMD17410933ST-11-0 (control) |
| SRS012273 (Healthy) | SAMN00791976 (Healthy) | SAMEA2581888 (HD-18) | CCMD18579000ST-11-0 (control) |
| SRS012902 (Healthy) | SAMN00791977 (Healthy) | SAMEA2581890 (HD-19) | CCMD18829815ST-11-0 (control) |
| SRS013158 (Healthy) | SAMN00791978 (Healthy) | SAMEA2581892 (HD-2) | CCMD18872694ST-21-0 (CRC) |
| SRS013215 (Healthy) | SAMN00791979 (Healthy) | SAMEA2581893 (HD-20) | CCMD19168690ST-11-0 (CRC) |
| SRS013476 (Healthy) | SAMN00792013 (Healthy) | SAMEA2581894 (HD-21) | CCMD19497912ST-21-0 (CRC) |
| SRS013521 (Healthy) | SAMN00792014 (Healthy) | SAMEA2581895 (HD-22) | CCMD21593359ST-11-0 (CRC) |
| SRS013687 (Healthy) | SAMN00792015 (Healthy) | SAMEA2581896 (HD-23) | CCMD21703880ST-11-0 (CRC) |
| SRS013800 (Healthy) | SAMN00792016 (Healthy) | SAMEA2581897 (HD-24) | CCMD22852639ST-11-0 (control) |
| SRS013951 (Healthy) | SAMN00792017 (Healthy) | SAMEA2581898 (HD-25) | CCMD23101338ST-21-0 (CRC) |
| SRS014235 (Healthy) | SAMN00792018 (T2D) | SAMEA2581900 (HD-26) | CCMD23541216ST-11-0 (control) |
| SRS014313 (Healthy) | SAMN00792019 (T2D) | SAMEA2581901 (HD-27) | CCMD25475945ST-11-0 (control) |
| SRS014459 (Healthy) | SAMN00792020 (T2D) | SAMEA2581902 (HD-28) | CCMD25511638ST-21-0 (CRC) |
| SRS016095 (Healthy) | SAMN00792021 (T2D) | SAMEA2581903 (HD-29) | CCMD25963797ST-21-0 (CRC) |
| SRS016267 (Healthy) | SAMN00792022 (T2D) | SAMEA2581904 (HD-3) | CCMD26625622ST-11-0 (control) |
| SRS016335 (Healthy) | SAMN00792023 (T2D) | SAMEA2581906 (HD-30) | CCMD26709089ST-21-0 (CRC) |
| SRS016495 (Healthy) | SAMN00792025 (T2D) | SAMEA2581907 (HD-31) | CCMD26925580ST-21-0 (CRC) |
| SRS016517 (Healthy) | SAMN00792031 (T2D) | SAMEA2581908 (HD-32) | CCMD27463710ST-11-0 (CRC) |
| SRS016585 (Healthy) | SAMN00792039 (T2D) | SAMEA2581910 (HD-34) | CCMD27867141ST-11-0 (control) |
| SRS016753 (Healthy) | SAMN00792045 (T2D) | SAMEA2581911 (HD-35) | CCMD28738636ST-11-0 (control) |
| SRS017103 (Healthy) | SAMN00792053 (T2D) | SAMEA2581913 (HD-37) | CCMD29706695ST-11-0 (control) |
| SRS017191 (Healthy) | SAMN00792059 (T2D) | SAMEA2581915 (HD-38) | CCMD30626189ST-11-0 (control) |
| SRS017307 (Healthy) | SAMN00792066 (T2D) | SAMEA2581916 (HD-39) | CCMD30627121ST-11-0 (control) |
| SRS017433 (Healthy) | SAMN00792069 (T2D) | SAMEA2581918 (HD-4) | CCMD31009081ST-11-0 (control) |
| SRS017521 (Healthy) | SAMN00792070 (T2D) | SAMEA2581919 (HD-40) | CCMD31134579ST-11-0 (control) |
| SRS018133 (Healthy) | SAMN00792071 (T2D) | SAMEA2581920 (HD-41) | CCMD32288175ST-11-0 (control) |
| SRS018313 (Healthy) | SAMN00792072 (T2D) | SAMEA2581921 (HD-42) | CCMD32965613ST-11-0 (control) |
| SRS018427 (Healthy) | SAMN00792073 (T2D) | SAMEA2581922 (HD-43) | CCMD34381688ST-21-0 (CRC) |
| SRS018575 (Healthy) | SAMN00792075 (T2D) | SAMEA2581924 (HD-44) | CCMD35081859ST-11-0 (control) |
| SRS018817 (Healthy) | SAMN00792076 (T2D) | SAMEA2581928 (HD-46) | CCMD35633353ST-11-0 (CRC) |
| SRS018984 (Healthy) | SAMN00792077 (T2D) | SAMEA2581929 (HD-47) | CCMD35801800ST-11-0 (control) |
| SRS019030 (Healthy) | SAMN00792078 (T2D) | SAMEA2581931 (HD-48) | CCMD35802077ST-21-0 (CRC) |
| SRS019161 (Healthy) | SAMN00792079 (T2D) | SAMEA2581932 (HD-49) | CCMD37575804ST-11-0 (control) |
| SRS019267 (Healthy) | SAMN00792080 (T2D) | SAMEA2581934 (HD-5) | CCMD38158721ST-11-0 (control) |
| SRS019397 (Healthy) | SAMN00792081 (T2D) | SAMEA2581935 (HD-50) | CCMD39157124ST-11-0 (control) |
| SRS019601 (Healthy) | SAMN00792082 (T2D) | SAMEA2581936 (HD-51) | CCMD39882286ST-11-0 (control) |
| SRS019685 (Healthy) | SAMN00792083 (T2D) | SAMEA2581937 (HD-52) | CCMD40701343ST-21-0 (CRC) |
| SRS019787 (Healthy) | SAMN00792087 (T2D) | SAMEA2581938 (HD-53) | CCMD41202658ST-11-0 (control) |
| SRS019968 (Healthy) | SAMN00792088 (T2D) | SAMEA2581939 (HD-54) | CCMD41521570ST-11-0 (control) |
| SRS020233 (Healthy) | SAMN00792089 (T2D) | SAMEA2581941 (HD-55) | CCMD42428553ST-21-0 (CRC) |
| SRS020328 (Healthy) | SAMN00792090 (T2D) | SAMEA2581943 (HD-56) | CCMD42956136ST-11-0 (CRC) |
| SRS020869 (Healthy) | SAMN00792091 (T2D) | SAMEA2581944 (HD-57) | CCMD45004878ST-11-0 (control) |
| SRS021484 (Healthy) | SAMN00792092 (T2D) | SAMEA2581946 (HD-58) | CCMD45812507ST-11-0 (control) |
| SRS021948 (Healthy) | SAMN00792093 (T2D) | SAMEA2581947 (HD-59) | CCMD45988061ST-21-0 (CRC) |
| SRS022071 (Healthy) | SAMN00792094 (T2D) | SAMEA2581949 (HD-6) | CCMD46222674ST-21-0 (CRC) |
| SRS022137 (Healthy) | SAMN00792095 (T2D) | SAMEA2581951 (HD-60) | CCMD46727384ST-11-0 (control) |
| SRS022609 (Healthy) | SAMN00792096 (T2D) | SAMEA2581952 (HD-61) | CCMD46947453ST-21-0 (CRC) |
| SRS023176 (Healthy) | SAMN00792097 (T2D) | SAMEA2581953 (HD-62) | CCMD48236717ST-21-0 (CRC) |
| SRS023526 (Healthy) | SAMN00792098 (T2D) | SAMEA2581954 (HD-63) | CCMD49025643ST-11-0 (control) |
| SRS023583 (Healthy) | SAMN00792099 (T2D) | SAMEA2581955 (HD-64) | CCMD49461418ST-11-0 (CRC) |
| SRS023829 (Healthy) | SAMN00792101 (T2D) | SAMEA2581956 (HD-65) | CCMD49942357ST-11-0 (control) |
| SRS023914 (Healthy) | SAMN00792102 (T2D) | SAMEA2581957 (HD-66) | CCMD50071957ST-21-0 (CRC) |
| SRS023971 (Healthy) | SAMN00792103 (T2D) | SAMEA2581958 (HD-67) | CCMD50300306ST-11-0 (control) |
| SRS024009 (Healthy) | SAMN00792104 (T2D) | SAMEA2581959 (HD-68) | CCMD50529145ST-11-0 (control) |
| SRS024075 (Healthy) | SAMN00792105 (T2D) | SAMEA2581960 (HD-69) | CCMD50538120ST-11-0 (control) |
| SRS024132 (Healthy) | SAMN00792106 (T2D) | SAMEA2581961 (HD-7) | CCMD51154251ST-11-0 (control) |
| SRS024331 (Healthy) | SAMN00792107 (T2D) | SAMEA2581962 (HD-70) | CCMD51228890ST-11-0 (control) |
| SRS024388 (Healthy) | SAMN00792108 (T2D) | SAMEA2581963 (HD-71) | CCMD51864103ST-21-0 (CRC) |
| SRS024435 (Healthy) | SAMN00792109 (T2D) | SAMEA2581964 (HD-72) | CCMD52117727ST-11-0 (CRC) |
| SRS024549 (Healthy) | SAMN00792110 (T2D) | SAMEA2581965 (HD-73) | CCMD52145360ST-11-0 (CRC) |
| SRS024625 (Healthy) | SAMN00792111 (T2D) | SAMEA2581966 (HD-74) | CCMD53508245ST-11-0 (control) |
| SRS042628 (Healthy) | SAMN00792112 (T2D) | SAMEA2581967 (HD-75) | CCMD53522274ST-11-0 (control) |
| SRS043001 (Healthy) | SAMN00792113 (T2D) | SAMEA2581968 (HD-76) | CCMD54057834ST-11-0 (control) |
| SRS043701 (Healthy) | SAMN00792114 (T2D) | SAMEA2581969 (HD-77) | CCMD56948710ST-11-0 (control) |
| SRS045004 (Healthy) | SAMN00792115 (T2D) | SAMEA2581970 (HD-78) | CCMD59540613ST-11-0 (control) |
| SRS045645 (Healthy) | SAMN00792116 (T2D) | SAMEA2581971 (HD-79) | CCMD59583015ST-11-0 (CRC) |
| SRS045713 (Healthy) | SAMN00792117 (T2D) | SAMEA2581972 (HD-8) | CCMD60079086ST-21-0 (CRC) |
| SRS047014 (Healthy) | SAMN00792118 (T2D) | SAMEA2581973 (HD-80) | CCMD63399960ST-21-0 (CRC) |
| SRS047044 (Healthy) |  | SAMEA2581974 (HD-81) | CCMD64503764ST-21-0 (CRC) |
| SRS048164 (Healthy) |  | SAMEA2581975 (HD-82) | CCMD64776337ST-21-0 (CRC) |
| SRS048870 (Healthy) |  | SAMEA2581976 (HD-83) | CCMD65222621ST-11-0 (CRC) |
| SRS049712 (Healthy) |  | SAMEA2581977 (HD-9) | CCMD65406197ST-11-0 (CRC) |
| SRS049900 (Healthy) |  | SAMEA2582018 (LD-1) | CCMD66848156ST-11-0 (control) |
| SRS049959 (Healthy) |  | SAMEA2582019 (LD-10) | CCMD67373733ST-11-0 (control) |
| SRS049995 (Healthy) |  | SAMEA2582021 (LD-11) | CCMD68973846ST-11-0 (control) |
| SRS050752 (Healthy) |  | SAMEA2582022 (LD-12) | CCMD71242853ST-11-0 (CRC) |
| SRS050925 (Healthy) |  | SAMEA2582023 (LD-13) | CCMD71915439ST-11-0 (CRC) |
| SRS051031 (Healthy) |  | SAMEA2582025 (LD-14) | CCMD72666896ST-11-0 (control) |
| SRS051882 (Healthy) |  | SAMEA2582026 (LD-15) | CCMD72690923ST-11-0 (CRC) |
| SRS052027 (Healthy) |  | SAMEA2582027 (LD-16) | CCMD72895164ST-21-0 (CRC) |
| SRS052697 (Healthy) |  | SAMEA2582028 (LD-17) | CCMD73128545ST-11-0 (control) |
| SRS053214 (Healthy) |  | SAMEA2582029 (LD-18) | CCMD74592084ST-11-0 (control) |
| SRS053398 (Healthy) |  | SAMEA2582031 (LD-19) | CCMD74930188ST-21-0 (CRC) |
| SRS054590 (Healthy) |  | SAMEA2582032 (LD-2) | CCMD75147712ST-11-0 (control) |
| SRS054956 (Healthy) |  | SAMEA2582034 (LD-20) | CCMD76222476ST-11-0 (CRC) |
| SRS056259 (Healthy) |  | SAMEA2582035 (LD-21) | CCMD76409700ST-11-0 (control) |
| SRS056519 (Healthy) |  | SAMEA2582037 (LD-22) | CCMD79349503ST-11-0 (CRC) |
| SRS058723 (Healthy) |  | SAMEA2582038 (LD-23) | CCMD79987997ST-21-0 (CRC) |
| SRS058770 (Healthy) |  | SAMEA2582039 (LD-24) | CCMD82866709ST-21-0 (CRC) |
| SRS062427 (Healthy) |  | SAMEA2582041 (LD-25) | CCMD85481373ST-11-0 (control) |
| SRS063985 (Healthy) |  | SAMEA2582043 (LD-26) | CCMD85661207ST-11-0 (control) |
| SRS075398 (Healthy) |  | SAMEA2582044 (LD-27) | CCMD86114146ST-21-0 (CRC) |
| SRS077730 (Healthy) |  | SAMEA2582047 (LD-28) | CCMD86707194ST-21-0 (CRC) |
| CSM67UBB (IBD) |  | SAMEA2582049 (LD-29) | CCMD87156761ST-21-0 (CRC) |
| CSM67UBH (IBD) |  | SAMEA2582050 (LD-3) | CCMD88272491ST-21-0 (CRC) |
| CSM67UGO (IBD) |  | SAMEA2582051 (LD-30) | CCMD89107682ST-11-0 (control) |
| CSM79HG5 (IBD) |  | SAMEA2582054 (LD-31) | CCMD89306485ST-11-0 (CRC) |
| CSM79HHO (IBD) |  | SAMEA2582057 (LD-32) | CCMD89643949ST-11-0 (control) |
| CSM79HHW (IBD) |  | SAMEA2582059 (LD-33) | CCMD89967135ST-11-0 (control) |
| CSM79HJA (IBD) |  | SAMEA2582060 (LD-34) | CCMD90311071ST-21-0 (CRC) |
| CSM79HJQ (IBD) |  | SAMEA2582061 (LD-35) | CCMD92404903ST-11-0 (CRC) |
| CSM79HK9 (IBD) |  | SAMEA2582062 (LD-36) | CCMD93344354ST-21-0 (CRC) |
| CSM79HOL (IBD) |  | SAMEA2582065 (LD-37) | CCMD93755960ST-11-0 (control) |
| CSM79HQX (IBD) |  | SAMEA2582066 (LD-38) | CCMD95431029ST-11-0 (CRC) |
| CSM79HRG (IBD) |  | SAMEA2582067 (LD-39) | CCMD95433940ST-21-0 (CRC) |
| CSM7KOLA (IBD) |  | SAMEA2582068 (LD-4) | CCMD95676152ST-11-0 (CRC) |
| CSM7KOMH (IBD) |  | SAMEA2582069 (LD-40) | CCMD96553385ST-21-0 (CRC) |
| CSM7KON2 (IBD) |  | SAMEA2582072 (LD-41) | CCMD98198513ST-11-0 (control) |
| CSM7KOPU (IBD) |  | SAMEA2582073 (LD-42) | CCMD98531134ST-11-0 (CRC) |
| CSM7KORK (IBD) |  | SAMEA2582074 (LD-43) | CCMD98702133ST-11-0 (control) |
| CSM7KOTQ (IBD) |  | SAMEA2582075 (LD-44) | CCMD99440714ST-11-0 (control) |
| CSM9X1ZC (IBD) |  | SAMEA2582076 (LD-45) | CCMD99929634ST-11-0 (control) |
| ESM718T7 (IBD) |  | SAMEA2582077 (LD-46) |  |
| ESM718T9 (IBD) |  | SAMEA2582078 (LD-47) |  |
| ESM718UH (IBD) |  | SAMEA2582080 (LD-48) |  |
| ESM7F5CB (IBD) |  | SAMEA2582082 (LD-49) |  |
| HSM5MD4O (IBD) |  | SAMEA2582084 (LD-5) |  |
| HSM67VDP (IBD) |  | SAMEA2582085 (LD-50) |  |
| HSM67VEK (IBD) |  | SAMEA2582086 (LD-51) |  |
| HSM67VFH (IBD) |  | SAMEA2582087 (LD-52) |  |
| HSM67VFJ (IBD) |  | SAMEA2582088 (LD-53) |  |
| HSM67VHW (IBD) |  | SAMEA2582089 (LD-54) |  |
| HSM6XRS6 (IBD) |  | SAMEA2582090 (LD-55) |  |
| HSM6XRST (IBD) |  | SAMEA2582092 (LD-56) |  |
| HSM6XRVW (IBD) |  | SAMEA2582094 (LD-57) |  |
| HSM7CYX2 (IBD) |  | SAMEA2582095 (LD-58) |  |
| HSM7CYXA (IBD) |  | SAMEA2582096 (LD-59) |  |
| HSM7J4J9 (IBD) |  | SAMEA2582097 (LD-6) |  |
| HSM7J4JP (IBD) |  | SAMEA2582099 (LD-60) |  |
| HSM7J4PU (IBD) |  | SAMEA2582100 (LD-61) |  |
| HSM7J4QF (IBD) |  | SAMEA2582101 (LD-62) |  |
| HSMA33KE (IBD) |  | SAMEA2582102 (LD-63) |  |
| HSMA33PN (IBD) |  | SAMEA2582103 (LD-64) |  |
| HSMA33QY (IBD) |  | SAMEA2582105 (LD-65) |  |
| HSMA33R7 (IBD) |  | SAMEA2582108 (LD-66) |  |
| MSM5LLDA (IBD) |  | SAMEA2582109 (LD-67) |  |
| MSM5LLER (IBD) |  | SAMEA2582111 (LD-68) |  |
| MSM5LLF6 (IBD) |  | SAMEA2582112 (LD-69) |  |
| MSM6J2II (IBD) |  | SAMEA2582113 (LD-7) |  |
| MSM6J2IM (IBD) |  | SAMEA2582114 (LD-70) |  |
| MSM6J2IO (IBD) |  | SAMEA2582116 (LD-71) |  |
| MSM79H69 (IBD) |  | SAMEA2582118 (LD-72) |  |
| MSM79HBN (IBD) |  | SAMEA2582119 (LD-73) |  |
| MSM7J16R (IBD) |  | SAMEA2582120 (LD-74) |  |
| MSM9VZEK (IBD) |  | SAMEA2582121 (LD-75) |  |
| MSMA26CX (IBD) |  | SAMEA2582122 (LD-76) |  |
| PSM6XBR1 (IBD) |  | SAMEA2582123 (LD-77) |  |
| PSM7J18E (IBD) |  | SAMEA2582124 (LD-78) |  |
| PSM7J18I (IBD) |  | SAMEA2582125 (LD-79) |  |
| PSMA2668 (IBD) |  | SAMEA2582126 (LD-8) |  |
| CSM5MCXR (IBD) |  | SAMEA2582127 (LD-80) |  |
| CSM67UBX (IBD) |  | SAMEA2582128 (LD-81) |  |
| CSM67UBZ (IBD) |  | SAMEA2582129 (LD-82) |  |
| CSM67UDR (IBD) |  | SAMEA2582130 (LD-83) |  |
| CSM7KOJS (IBD) |  | SAMEA2582131 (LD-84) |  |
| CSM7KOJY (IBD) |  | SAMEA2582132 (LD-85) |  |
| CSM7KORS (IBD) |  | SAMEA2582133 (LD-86) |  |
| CSM9X1Y5 (IBD) |  | SAMEA2582134 (LD-87) |  |
| CSM9X21N (IBD) |  | SAMEA2582136 (LD-88) |  |
| CSM9X22U (IBD) |  | SAMEA2582137 (LD-89) |  |
| CSM9X23N (IBD) |  | SAMEA2582138 (LD-9) |  |
| ESM5ME9U (IBD) |  | SAMEA2582139 (LD-90) |  |
| ESM5MEBS (IBD) |  | SAMEA2582140 (LD-91) |  |
| HSM6XRTS (IBD) |  | SAMEA2582141 (LD-92) |  |
| HSM7CZ1Z (IBD) |  | SAMEA2582142 (LD-93) |  |
| HSM7J4HS (IBD) |  | SAMEA2582143 (LD-94) |  |
| HSM7J4K4 (IBD) |  | SAMEA2582144 (LD-95) |  |
| HSM7J4M4 (IBD) |  | SAMEA2582145 (LD-96) |  |
| HSM7J4ME (IBD) |  | SAMEA2582146 (LD-97) |  |
| HSM7J4NO (IBD) |  | SAMEA2582147 (LD-98) |  |
| HSM7J4PE (IBD) |  |  |  |
| HSMA33OX (IBD) |  |  |  |
| MSM6J2HD (IBD) |  |  |  |
| MSM6J2OL (IBD) |  |  |  |
| MSM79H7G (IBD) |  |  |  |
| MSM79HDC (IBD) |  |  |  |
| MSM79HEA (IBD) |  |  |  |
| MSM79HF5 (IBD) |  |  |  |
| MSM9VZF3 (IBD) |  |  |  |
| MSM9VZNR (IBD) |  |  |  |
| MSM9VZNX (IBD) |  |  |  |
| MSM9VZOU (IBD) |  |  |  |
| MSM9VZP1 (IBD) |  |  |  |
| MSMA26AZ (IBD) |  |  |  |
| MSMA26EZ (IBD) |  |  |  |
| PSM7J12F (IBD) |  |  |  |
| PSM7J169 (IBD) |  |  |  |
| PSM7J1AU (IBD) |  |  |  |
| PSM7J1B7 (IBD) |  |  |  |
| PSM7J1BB (IBD) |  |  |  |
| PSMA264Q (IBD) |  |  |  |
| PSMA2671 (IBD) |  |  |  |
| PSMB4MBI (IBD) |  |  |  |

**Supplementary Table S2.** Accuracy and execution time of different MLP models with different number of layers and nodes. For the input dataset, genome-level relative abundance of IBD dataset using 30 reference samples were used.

Accuracy

| **No. of**  **hidden layers** | **1** | **2** | **3** | **4** | **5** | **6** | **7** | **8** | **9** | **10** |
| --- | --- | --- | --- | --- | --- | --- | --- | --- | --- | --- |
| 20 nodes each | 0.915 | 0.93 | 0.925 | 0.915 | 0.93 | 0.925 | 0.935 | 0.925 | 0.92 | 0.925 |
| 50 nodes each | 0.935 | 0.94 | 0.945 | 0.93 | 0.935 | 0.9 | 0.925 | 0.93 | 0.93 | 0.935 |
| 100 nodes each | 0.925 | 0.955 | 0.945 | 0.95 | 0.935 | 0.9 | 0.935 | 0.94 | 0.915 | 0.925 |

Execution time (seconds)

| **No. of**  **hidden layers** | **1** | **2** | **3** | **4** | **5** | **6** | **7** | **8** | **9** | **10** |
| --- | --- | --- | --- | --- | --- | --- | --- | --- | --- | --- |
| 20 nodes each | 793.1 | 1059.2 | 1296.6 | 1490.2 | 1509.9 | 1954.3 | 2209.9 | 2429.7 | 2597.9 | 2801.9 |
| 50 nodes each | 909.3 | 1156.8 | 1369.6 | 1509.4 | 1758.2 | 2062.8 | 2072.2 | 2366.7 | 2686.4 | 2830.5 |
| 100 nodes each | 1135.9 | 1341.8 | 1478.8 | 1700.5 | 1935.2 | 2023.1 | 2289.7 | 2537.8 | 2667.3 | 2795.6 |

Model structures (accuracy and execution time)

| **No. of**  **hidden layers** | **Structures of hidden layers** | **Accuracy** | **Time**  **(seconds)** |
| --- | --- | --- | --- |
| 3 layers | 200 – 100 – 50 | 0.93 | 1318.1 |
| 4 layers | 200 – 150 – 100 – 50 | 0.92 | 1436.1 |
| 5 layers | 200 – 150 – 100 x 2 – 50 | 0.93 | 1540.0 |
| 6 layers | 200 – 150 x 2 – 100 x 2 – 50 | 0.94 | 1779.3 |
| 7 layers | 200 – 150 x 2 – 100 x 2 – 50 x 2 | 0.905 | 1829.4 |
| 8 layers | 200 x 2 – 150 x 2 – 100 x 2 – 50 x 2 | 0.935 | 2062.7 |
| 9 layers | 200 x 2 – 150 x 2 – 100 x 3 – 50 x 2 | 0.93 | 2142.3 |
| 10 layers | 200 x 2 – 150 x 3 – 100 x 3 – 50 x 2 | 0.93 | 2281.2 |
| 11 layers | 200 x 2 – 150 x 3 – 100 x 3 – 50 x 3 | 0.92 | 3380.0 |
| 12 layers | 200 x 3 – 150 x 3 – 100 x 3 – 50 x 3 | 0.93 | 3454.1 |
| 13 layers | 200 x 3 – 150 x 3 – 100 x 4 – 50 x 3 | 0.925 | 3577.4 |
| 14 layers | 200 x 3 – 150 x 4 – 100 x 4 – 50 x 3 | 0.895 | 3930.2 |
| 15 layers | 200 x 3 – 150 x 4 – 100 x 4 – 50 x 4 | 0.89 | 4126.2 |

**Supplementary Table S3.** The performance with four different model architectures

| **Features** | | **IBD** | | | **T2D** | | | **LC** | | | **CRC** | | | |
| --- | --- | --- | --- | --- | --- | --- | --- | --- | --- | --- | --- | --- | --- | --- |
|  |  | **P** | **R** | **A** | **P** | **R** | **A** | **P** | **R** | **A** | **P** | **R** | **A** |  |
| Taxonomy | Phylum | 0.69 (0.02) | 0.71 (0.05) | 0.70 (0.02) | 0.66 (0.02) | 0.80 (0.05) | 0.58 (0.04) | 0.77 (0.02) | 0.57 (0.01) | 0.68 (0.01) | 0.57 (0.03) | 0.41 (0.05) | 0.55 (0.02) |  |
|  | Class | 0.70 (0.01) | 0.69 (0.03) | 0.70 (0.01) | 0.69 (0.01) | 0.76 (0.01) | 0.60 (0.01) | 0.81 (0.02) | 0.77 (0.03) | 0.78 (0.02) | 0.55 (0.04) | 0.52 (0.04) | 0.55 (0.04) |  |
|  | Order | 0.75 (0.02) | 0.70 (0.02) | 0.74 (0.01) | 0.69 (0.01) | 0.76 (0.05) | 0.60 (0.03) | 0.79 (0.00) | 0.78 (0.02) | 0.77 (0.00) | 0.57 (0.02) | 0.58 (0.03) | 0.58 (0.02) |  |
|  | Family | 0.84 (0.02) | 0.82 (0.03) | 0.83 (0.02) | 0.73 (0.03) | 0.76 (0.05) | 0.65 (0.04) | 0.78 (0.01) | 0.79 (0.02) | 0.77 (0.01) | 0.62 (0.03) | 0.64 (0.04) | 0.63 (0.03) |  |
|  | Genus | 0.87 (0.02) | 0.87 (0.01) | 0.87 (0.01) | 0.76 (0.01) | 0.77 (0.05) | 0.68 (0.03) | 0.83 (0.01) | 0.82 (0.01) | 0.81 (0.01) | 0.66 (0.03) | 0.66 (0.01) | 0.66 (0.02) |  |
|  | Species | 0.91 (0.01) | 0.87 (0.02) | 0.89 (0.01) | 0.75 (0.01) | 0.82 (0.01) | 0.69 (0.01) | 0.86 (0.01) | 0.78 (0.02) | 0.81 (0.00) | 0.71 (0.01) | 0.67 (0.04) | 0.70 (0.00) |  |
| Genomic contigs  (3 runs) | 2 Refs. | 0.89 (0.03) | 0.85 (0.05) | 0.87 (0.04) | 0.72 (0.02) | 0.80 (0.03) | 0.65 (0.01) | 0.71 (0.07) | 0.73 (0.07) | 0.70 (0.07) | 0.62 (0.02) | 0.54 (0.02) | 0.61 (0.02) |  |
|  | 10 Refs. | 0.88 (0.06) | 0.88 (0.03) | 0.88 (0.05) | 0.73 (0.00) | 0.82 (0.03) | 0.67 (0.01) | 0.69 (0.08) | 0.70 (0.10) | 0.68 (0.09) | 0.66 (0.05) | 0.58 (0.04) | 0.64 (0.04) |  |
|  | 20 Refs. | 0.89 (0.04) | 0.84 (0.07) | 0.87 (0.05) | 0.75 (0.01) | 0.83 (0.06) | 0.70 (0.03) | 0.79 (0.01) | 0.78 (0.02) | 0.77 (0.01) | 0.72 (0.05) | 0.65 (0.02) | 0.70 (0.04) |  |
|  | 30 Refs. | 0.94 (0.01) | 0.90 (0.02) | 0.92 (0.01) | 0.75 (0.02) | 0.81 (0.04) | 0.68 (0.03) | 0.79 (0.04) | 0.78 (0.04) | 0.77 (0.03) | 0.82 (0.02) | 0.69 (0.04) | 0.77 (0.02) |  |
|  | 40 Refs. | 0.94 (0.01) | 0.92 (0.01) | 0.93 (0.01) | 0.77 (0.02) | 0.85 (0.04) | 0.72 (0.04) | 0.81 (0.02) | 0.80 (0.03) | 0.79 (0.02) | 0.84 (0.02) | 0.75 (0.01) | 0.81 (0.01) |  |
| Functional | | 0.77 (0.03) | 0.85 (0.05) | 0.80 (0.03) | 0.74 (0.01) | 0.86 (0.05) | 0.70 (0.01) | 0.74 (0.02) | 0.88 (0.03) | 0.77 (0.02) | 0.98 (0.01) | 0.90 (0.02) | 0.94 (0.01) |  |
| All combined | | 0.97 (0.01) | 0.98 (0.01) | 0.98 (0.00) | 0.80 (0,.02) | 0.86 (0.02) | 0.76 (0.03) | 0.86 (0.01) | 0.83 (0.02) | 0.84 (0.01) | 0.99 (0.01) | 0.94 (0.01) | 0.97 (0.01) |  |

*For each experiment, LOOCV was used for calculating precision, recall and accuracy. Five iterations were performed and standard deviations are shown between round brackets. For genomic features, three runs were conducted selecting reference samples independently. P: precision; R: recall; and A: accuracy.

**Supplementary Table S4.** Number of contigs regarding the binning and the number of representatives (one of three runs)

| **Dataset** | **Binning** | **2 Refs.** | **10 Refs.** | **20 Refs.** | **30 Refs.** | **40 Refs.** |
| --- | --- | --- | --- | --- | --- | --- |
| IBD | Without | 4,615 | 31,180 | 71,177 | 89,868 | 130,812 |
|  | With | 13 | 89 | 151 | 197 | 268 |
| T2D | Without | 8,238 | 35,063 | 68,817 | 95,281 | 145,512 |
|  | With | 50 | 107 | 195 | 259 | 366 |
| LC | Without | 2,722 | 18,330 | 37,119 | 66,685 | 92,966 |
|  | With | 22 | 74 | 121 | 179 | 235 |
| CRC | Without | 9,816 | 44,605 | 78,397 | 145,131 | 174,124 |
|  | With | 69 | 173 | 257 | 402 | 480 |

**Supplementary Table S5.** Running time for running LOOCV with various learning models of T2D dataset using taxonomic, genomic, and functional features. For genomic features, 40 reference samples were chosen out of 148 samples (control:47, disease: 101).

| **No.** | **Base model** | **Running time**  **(mm:ss)** |
| --- | --- | --- |
| 1 | Random forest | 00:34 |
| 2 | XGBoost | 10:37 |
| 3 | Principal component regression | 01:44 |
| 4 | Penalized regression (lasso) | 00:12 |
| 5 | Support vector machine (rbf kernel) | 00:22 |
| 6 | Ensemble (PCR, lasso, SVM) | 01:45 |
| 7 | Deep neural network (multimodal) | 145:44 |
